# Evaluation of hemodynamically significant pericardial effusion by analysis of cardiac chambers volume by computed tomography

**DOI:** 10.1259/bjr.20220106

**Published:** 2022-10-11

**Authors:** Yoav Granot, Zach Rozenbaum, Hila Yashar, Tamar Shalmon, Shlomo Berliner, Galit Aviram

**Affiliations:** ^1^ Departments of Cardiology, School of MedicineTel Aviv University, Tel Aviv, Israel; ^2^ Departments of Radiology, Tel Aviv Medical Center, Tel Aviv, Israel; ^3^ Departments of Internal Medicine, School of Medicine, Tel Aviv University, Tel Aviv, Israel

## Abstract

**Objective:**

Pericardial effusion may present clinically as pleuritic chest pain, dyspnea, or hemodynamic compromise and is a frequent finding in computerized tomographic pulmonary angiography (CTPA) exams. We hypothesized that CTPA-based analysis of the cardiac chamber volumes can be used to predict the hemodynamic significance of pericardial effusion (HsPE) as compared with echocardiography.

**Methods:**

Retrospective analysis of consecutive patients who underwent CTPA and echocardiography between January 2009 and November 2017 that ruled-out acute pulmonary embolism was included. Differences in cardiac chamber volumes were investigated in correlation to echocardiographic evidence of HsPE.

**Results:**

The final cohort included 208 patients, of whom 22 (11%) were diagnosed with HsPE. The HsPE patients had much smaller right cardiac chamber volumes (Median 78.8 ml (IQR 72.4–89.1)) than patients without HsPE (Median 115.1 ml (IQR 87.4–150). A decision tree for the prediction of HsPE showed multiple cutoff values. Right atrium (RA) volume had the best accuracy (area under the curve 0.851, 95% confidence interval 0.776–0.925, *p* < .001) for predicting the presence of HsPE. An RA volume ≤86 ml yielded a sensitivity of 95.5%, a specificity of 64%, and a NPV of 99.2% for the presence of HsPE.

**Conclusion:**

CTPA-based volumetric information with focus on the RA volume may help predict the presence of HsPE.

**Advances in knowledge:**

Pericardial effusion is a frequent finding in CTPA exams. Our study shows that CTPA-based volumetric information can predict the presence of HsPE with RA volume as the best indicator.

## Introduction

A computed tomography pulmonary angiogram (CTPA) is currently the modality of choice for the definitive diagnosis of acute pulmonary embolism (APE)^1^ The nonspecific symptoms associated with APE (shortness of breath, exercise intolerance, and chest discomfort), that are prevalent in other etiologies, leads to only 10–20% of patients undergoing CTPA with confirmed with APE.^2–4^ Pericardial effusion is present in around 5% of CTPAs.^5^ Clinical significant effusion are most often caused by neoplastic, infectious (most commonly tuberculosis), and uremic. The clinical implication of the effusion is related not only to the size but also to the rate of accumulation. Effusions that develop slowly can be remarkably asymptomatic, while rapidly accumulating smaller effusions can present with tamponade.^6,7^ Clinical significant pericardial effusion occurs when the increase in pericardial pressure by the fluid impairs diastolic filling and thus decreases stroke volume and cardiac output. Although patients could be asymptomatic, many experience dyspnea, chest pain weakness, and in its most severe manifestation present with hemodynamic compromise.^8^ The most important aspect of the early evaluation of patients with pericardial effusion is the hemodynamic impact of the fluid, and echocardiography is the methodology of choice for detecting hemodynamic changes before the occurrence of a clinical cardiac tamponade.^1,9^

Several reports have shown non-specific imaging CT findings of cardiac tamponade, including coronary sinus compression, inferior vena cava (IVC) dilatation, and reflux of contrast material into the IVC.^10–13^ Ohta et al reported that the combination of right ventricular outflow tract compression, IVC long and short axis diameter ratio, and effusion size had a sensitivity of only 81% in predicting the presence of cardiac tamponade.^14^ Their study did not include normotensive patients who were in a pre-tamponade state, which can be diagnosed by the presence of hemodynamically significant pericardial effusion (HsPE) on echocardiography.

Since early diagnosis of an impending tamponade could lead to improved outcome, the aim of the present study was to assess the diagnostic value of automated volumetric analysis of the cardiac chambers based on CTPA images for the identification of patients with HsPE.

## Material and methods

This study is a retrospective analysis performed in a single university-affiliated tertiary-care hospital. It was reviewed and approved by the Institutional Review Board with a waiver of informed consent.

### Study population

The hospital’s database was used to identify consecutive patients who underwent CTPA between January 2009 and November 2017 and for whom echocardiography results were available. The electronic medical records of the patients were used to retrieve radiologic information as well as clinical information (age, gender, and prior medical condition based on ICD-9 coding in the hospital electronic records) and echocardiographic data. The inclusion criteria were inpatients older than 18 years of age with evidence of pericardial effusion on the CTPA examination. Patients whose CTPA examination was technically inadequate for volumetric analysis were excluded.

### CT image acquisition

All patients were scanned by a multidetector CT scanner (Brilliance 64 or iCT 256; Philips Medical Systems, Cleveland, OH, USA) with 64 or 128 × 2 detector rows. The reconstructed slice thickness was 1.0 mm with increments of 0.5 mm. Scans were acquired according to our routine non-electrocardiographic-gated CTPA protocol with contrast injections of 70–100 ml of iodinated contrast material at a concentration of 300 mg iodine per mL (Ultravist, Schering, Berlin, Germany) and at rates of 3–4 mL/second. To optimize visualization of the pulmonary arteries, an automated bolus-tracking technique was used with a region of interest placed within the main pulmonary artery. Scanning began 5 s after reaching a threshold of 100 Housefield Units at the region of interest. All scans were obtained in a caudal-cranial direction during a single breath-hold.

### CT assessment

Automated volumetric measurements were obtained retrospectively for research purposes by a fully automatic algorithm (Pulmonary Arterial Analysis, Extended Brilliance Workplace Portal v. 7, Philips Health Care, Best, The Netherlands) that adapts an anatomic model of the heart chambers to the CT image volume.^15–18^ The volume of each chamber was automatically calculated with additional computation of the ratios between the various cardiac chambers’ volumes: right ventricle (RV) volume to left ventricle (LV) volume (ventricular volume ratio, VVR), and right atrial (RA) volume to left atrial (LA) volume (atrial volume ratio, AVR). Each exam was reviewed by an expert radiologist or cardiologist to assess the quality of the volume segmentation, and exams with inaccurate segmentation (*n* = 9) were excluded (S.Z.A, A.B, Z.R, Y.G, G.A., with 6, 3, 4, 3 and 15 years of experience, respectively). When height and weight were available, each chamber’s volume was adjusted to the body surface area and reported as the patient’s index volume.

### Doppler echocardiography

Pericardial effusion was assessed by a comprehensive two-dimensional and Doppler echocardiographic study with multiple windows during the same examination according to the American Society of Echocardiography (ASE) clinical recommendations.^19^ They included evaluation of the IVC and hepatic veins, evidence of right heart diastolic chamber collapse, increased respiratory variation in mitral inflow and left ventricular outflow tract outflow, and septal bounce. Patient were diagnosed as having HsPE if their echocardiographic findings were indicative, as per ASE 2013 consensus statement..

### Statistical analysis

Categorical variables were reported as numbers and percentages, and continuous variables were reported as means ± standard deviation or medians with interquartile range. Continuous variables were evaluated for normal distribution with histograms and Q-Q plots. The Mann-Whitney test was applied to compare continuous variables between groups, and the Chi-square test was used to compare categorical variables. Each continuous cardiac chamber volume was divided into categories by means of a chi-squared automatic interaction detection model or a classification and regression model with a maximum tree depth of 3 levels, minimum of 50 cases in parent node and minimum of 20 cases in child node. The analysis selects the best predictor for splitting the data into child nodes according with association with the presence of HsPE. A *P* value was given for each branch. To increase the validity of the decision tree, a fivefold cross-validation procedure was applied with similar cutoff points. The discrimination ability was evaluated with the area under the receiver operating characteristic curve. All statistical tests were two-sided, and a *P*-value of <.05 was considered statistically significant. SPSS software was used for all statistical analysis (IBM SPSS statistics, version 25, Armnok, NY, USA, 2017).

## Results

### Patient characteristics

The CTPA examination of nine patients was technically inadequate for volumetric analysis and they were excluded. Our final cohort included 208 patients, 106 patients (51%) with small while pericardial effusion, and 102 (49%) with moderate or large based upon CTPA assessment. 70% of the patients underwent echocardiography on the same 24 h as the CTPA exam. Twenty-two (11%) patients had HsPE per echocardiography, and 19 (9%) of them underwent urgent pericardiocentesis during their index hospitalization.

Table 1 provides the patients’ characteristics according to the presence of HsPE. Those with HsPE were younger, with a median age of 58.6 (IQR 47–72.7) years compared to 71 (57.5–79.2) years for the entire cohort. In addition, patients with HsPE were more likely to have a history of active malignancy (11 [50%] versus 35 [19%], respectively, *p* = .002), while those without HsPE were more likely to have a history of chronic lung disease (35 [19%] versus 0 [0%], *p* = .014), ischemic heart disease (46 [25%] versus 1 [5%], *p* = .021), atrial fibrillation (36 [19%] versus 0 [0%], *p* = .012), and chronic heart failure (43 [23%] versus 1 [5%], *p* = .03).

**Table 1.** Patients’ clinical Characteristics and Computerized Tomographic Measurements According to the Presence of HsPE

Variable	All patients (*n* = 208)	Hemodynamically insignificant effusion (*n* = 186)	Hemodynamically significant effusion (*n* = 22)	*P*-Value
Age, ya	71 (57.5–79.2)	71.6 (59.6–80.2)	58.6 (47–72.7)	.006
Male sex	90 (43)	83 (45)	7 (32)	.252
Lung disease	35 (17)	35 (19)	0	.014
Ischemic heart disease	47 (23)	46 (25)	1 (5)	.021
Atrial fibrillation	36 (17)	36 (19)	0	.012
CHF	44 (21)	43 (23)	1 (5)	.03
Active malignancy	46 (22)	35 (19)	11 (50)	.002
Hypertension	113 (54)	105 (57)	8 (36)	.074
Hyperlipidemia	67 (32)	62 (33)	5 (23)	.314
Active smoker	28 (14)	24 (13)	4 (18)	.341
Chronic renal failure	7 (3)	6 (3)	1 (5)	.548
Diabetes mellitus	49 (24)	47 (25)	2 (9)	.091
LV volume	70.1 (53.2–94.1)	72 (55.9–95.2)	45.9 (36–70.5)	< .001
LA volume	78.2 (55–112.5)	82.3 (58.6–115.9)	50.3 (37–57.1)	< .001
RV volume	110.4 (82.4–147.7)	115.1 (87.4–150)	78.8 (72.4–89.1)	< .001
RA volume	91 (68.9–129.6)	96.4 (73.1–135.3)	56.5 (50.9–75.4)	< .001
LV volume index	39.9 (30.4–52.7)	40.8 (32.5–53.4)	27.1 (22.8–39.6)	< .001
LA volume index	44.8 (31.4–65)	48 (33.4–66.5)	27.9 (22.1–32.7)	< .001
RV volume index	61.6 (48.3–83.3)	65.3 (50.6–86.3)	44.4 (37.9–50.2)	< .001
RA volume index	50.4 (39.4–76.5)	56 (41.4–80.1)	33.1 (28.8–41.6)	< .001
VVR	2.7 (2.2–3.6)	2.8 (2.2–3.7)	2.5 (2–3.5)	.361
AVR	2 (1.5–2.8)	2.1 (1.5–2.8)	1.9 (1.6–2.5)	.495
Effusion size by CT, n (%)				
Small	106 (51)	105 (56)	1 (5)	< .001
Moderate or greater	102 (49)	81 (44)	21 (95)	

AVR, atrium volume ratio; CT, computerized tomography; HsPE, hemodynamic significant pericardial effusion; LA, left atrium; LV, left ventricle; RA, right atrium; RV, right ventricle; VVR, ventricular volume ratio.

Volume is given in ml, and volume index is represented in ml/m².

a Median and interquartile range. All other values represent the number of patients and percentages.

### Volumetric analysis

**Figure 1.**
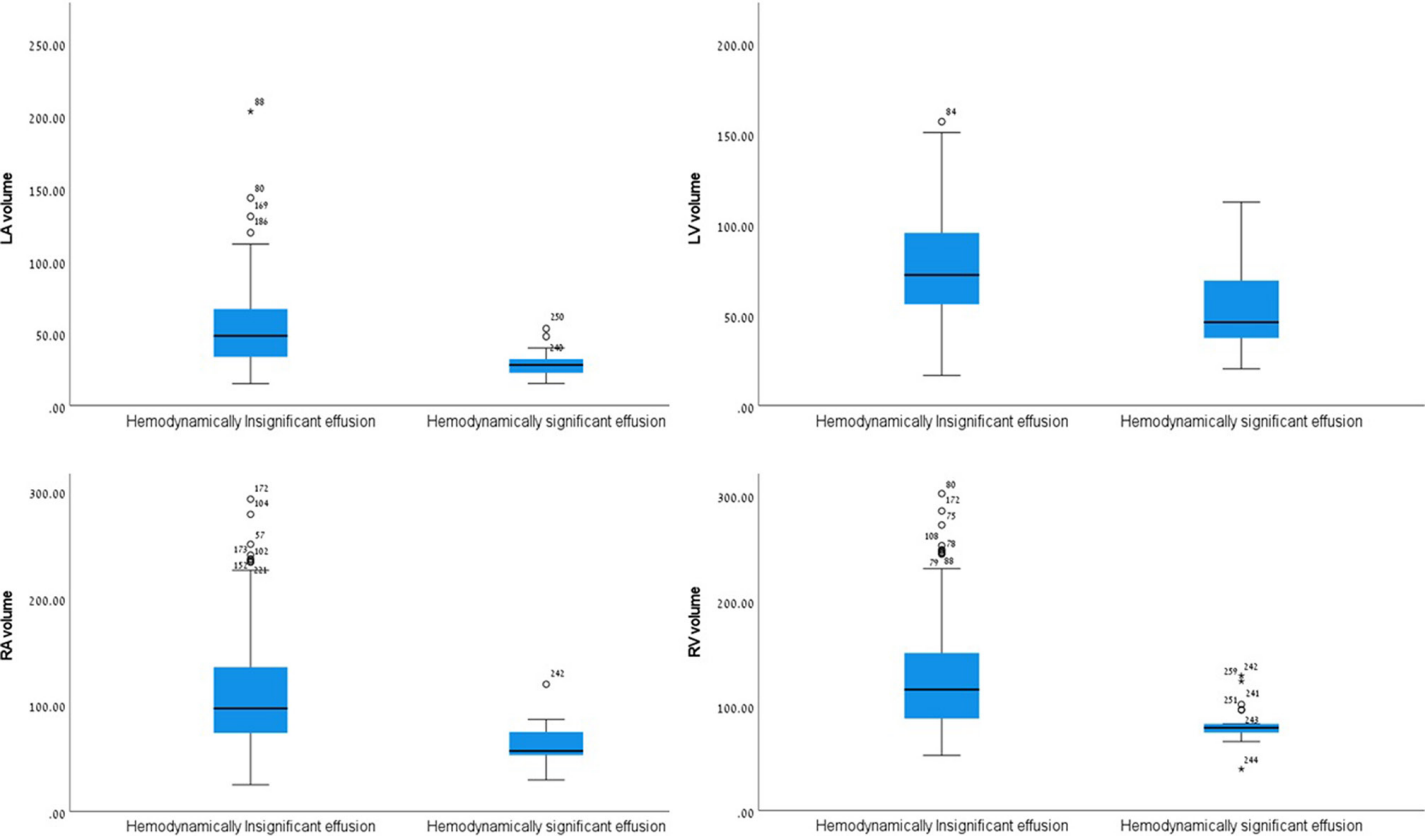
Box plots of the (**a**) left ventricle volume, (**b**) left atrium volume, (**c**) right ventricle volume, and (**d**) right atrium volume in patients with and without HsPE

**Figure 2.**
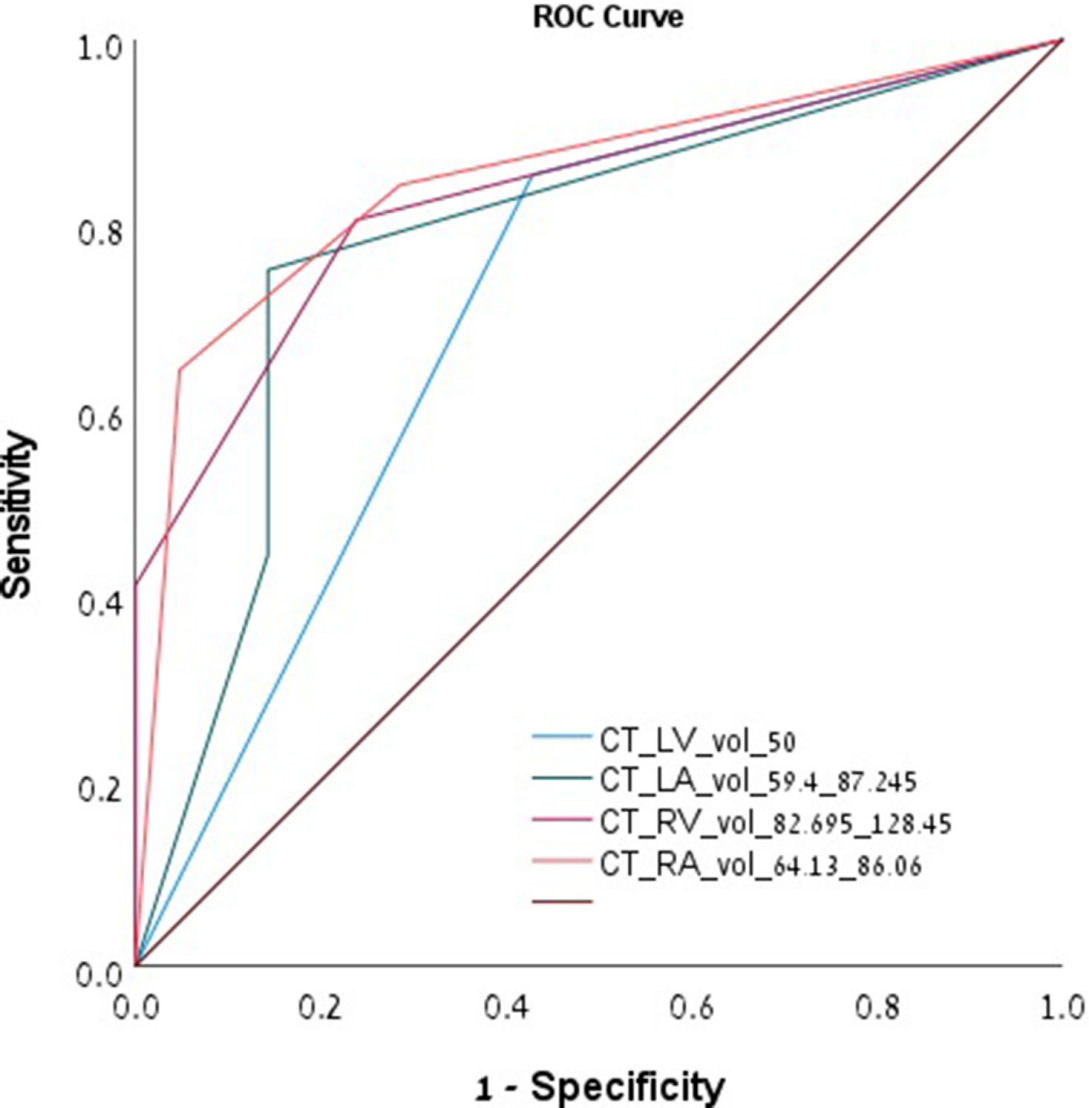
ROC curves for the LV, LA, RV, and RA cutoff values for the prediction of HsPE.

Table 1 lists the CT measurements of the cardiac chamber volumes according to the presence of HsPE. Patients with HsPE had a smaller volumes of all four cardiac chambers. Neither VVR nor AVR differed significantly between groups. Figure 1 illustrates the distribution of the various cardiac chamber volumes between patients with and without HsPE. A classification trees analysis revealed cutoff values for each cardiac chamber that were predictive for the presence of HsPE, and Table 2 shows their distribution and the accuracy of their application to identify patients with HsPE (area under the curve [AUC], *P* value). The areas under the ROC curves for the LV, LA, RV, and RA cutoff values were 0.713 (95%CI: 0.582–0.844, *p* = .001), 0.782 (95%CI: 0.672–0.892, *p* < .001), 0.833 (95%CI: 0.76–0.905, *p* < .001), and 0.851 (95%CI: 0.776–0.925, *p* < .001), respectively (Figure 2).

**Table 2.** Distribution of Various CT Measurements According to the Presence of HsPE and the Accuracy of the Measurements in Discriminating (Area under the Curve, *P* Value), Based on the Thresholds of the Classification Trees

Variable	Hemodynamically insignificant effusion (*n* = 186)	Hemodynamically significant effusion (*n* = 22)	AUC	*P* value
LV volume			0.713 (0.582–0.844)	.001
≤50	27 (15)	13 (59)		
>50	158 (85)	9 (41)		
LA volume			0.782 (0.672–0.892)	<.001
≤59.4	47 (25)	19 (86)		
59.4–87.25	57 (31)	0		
>87.25	82 (44)	3 (14)		
RV volume			0.833 (0.760–0.905)	<.001
≤82.7	36 (19)	16 (76)		
82.7–128.5	73 (39)	5 (24)		
>128.5	77 (41)	0		
RA volume			0.851 (0.776–0.925)	<.001
≤64.1	29 (16)	16 (73)		
64.1–86.1	37 (20)	5 (23)		
>86.1	120 (65)	1 (5)		

HsPE, hemodynamically significant pericardial effusion; LA, left atrium; LV, left ventricle; RA, right atrium; RV, right ventricle.

Volume is given in ml.

Number of patients and percentages.

Table 3 displays the odds ratio (OR) of each of the cardiac chamber volume cutoff values for the presence of HsPE. Patients with an LV volume ≤50 ml had an OR of 8.5 (95%CI 3.3–21.7, *p* < .001) to sustain a HsPE compared to all other patients. Patients with an LA volume ≤59.4 ml had an OR of 11 (95%CI 3.1–39.3, *p* < .001) compared with patients with LA volumes above 87.3 ml. Patients with an RV volume ≤82.7 ml had an OR of 6.5 (95%CI 2.2–19.1, *p* < .001) compared with patients with an RV volume between 82.7 and 128.5 ml. Since no patient with an RV volume >130 ml had HsPE, the OR could not be calculated for that cutoff. An RA volume ≤64.1 ml had an OR of 66.2 (95%CI 8.4–520, *p* < .001) compared with an RA volume >86.1. There was an OR of 16.2 (95%CI 1.8–143.2, *p* = .012) for an RV volume between 64.1 and 86.1 ml compared with an RA >86.1 ml. The sensitivity, specificity, PPV, NPV, and accuracy of each cutoff value for the diagnosis of HsPE are presented in Table 4. Of note, an RA volume ≤86.1 ml had a sensitivity of 95.5% and a specificity of 64%, with an NPV of 99.2% for the diagnosis of HsPE.

**Table 3.** Univariate Analyses for the Prediction of Hemodynamically

Significant Pericardial Effusion Chamber volume cutoff	Odds ratio (95% confidence interval)	*P* value
LV volume ≤50	8.5 (3.3–21.7)	<.001
LA volume ≤59.4 versus >87.3	11 (3.1–39.3)	<.001
RV volume ≤82.7 *vs* 82.7-128.5	6.5 (2.2–19.1)	<.001
RA volume ≤64.1 versus >86.1	66.2 (8.4–520)	<.001
RA volume 64.1–86.1 versus >86.1	16.2 (1.8–143.2)	.012

LA, left atrium; LV, left ventricle; RA, right atrium; RV, right ventricle.

Volume is given in ml.

**Table 4.** The Sensitivity, Specificity, Positive Predictive Value, Negative Predictive Value, and Accuracy of Each Cutoff Value for the Diagnosis of Hemodynamically Significant Pericardial Effusion

Variable	Sensitivity	Specificity	PPV	NPV	Accuracy
LV volume ≤50	59.1	85.4	32.5	94.6	82.6
LA volume ≤59.4	86.4	74.7	28.8	97.9	76
LA volume ≤87.2	86.4	44.1	15.4	96.5	48.6
RV volume ≤82.7	76.2	80.6	30.8	96.8	80.2
RV volume ≤128.5	100	41.4	16.2	100	47.3
RA volume ≤64.1	72.7	84.4	35.6	96.3	83.2
RA volume ≤86.1	95.5	64	23.9	99.2	67.3

LA, left atrium; LV, left atrium; NPV, negative predictive value; PPV, positive predictive value; RA, right atrium; RV, right ventricle.

Volumes are given in ml.

**Figure 3.**
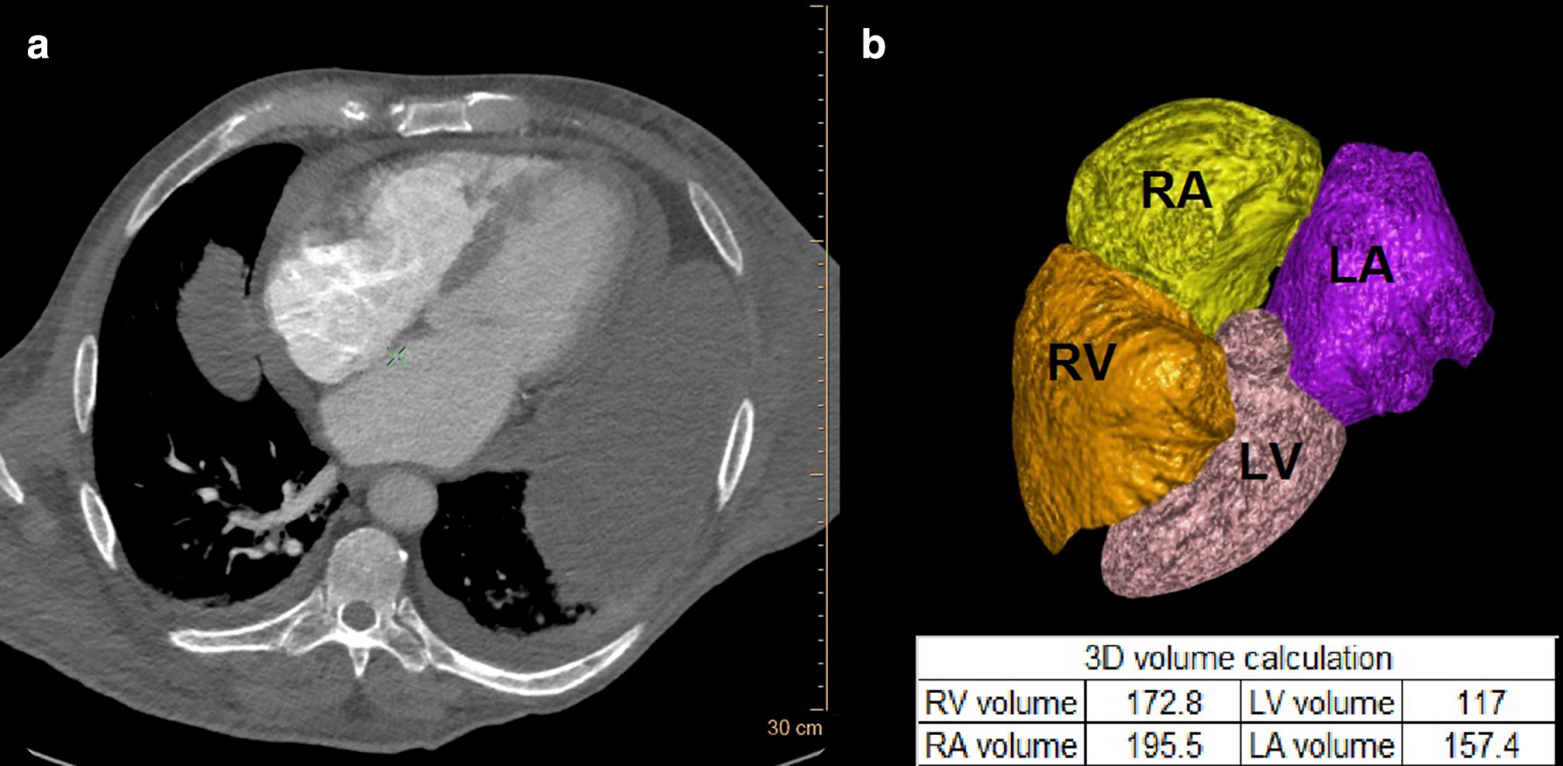
Representative case. Volumetric analysis of the CT pulmonary angiography (CTPA) of a 77-year-old male who presented to the emergency department with shortness of breath and clinical signs of right heart failure. CTPA ruled out pulmonary embolism, but there was a large pericardial effusion. His volumetric analysis was LV volume 117 ml, LA volume 157.4 ml, RV volume 172.8 ml, and RA volume 195.5. Echocardiography showed a moderate pericardial effusion with no signs of hemodynamic significance. He was provisionally diagnosed as having combined diastolic heart failure with COPD and appropriate treatment led to significant improvement.

**Figure 4.**
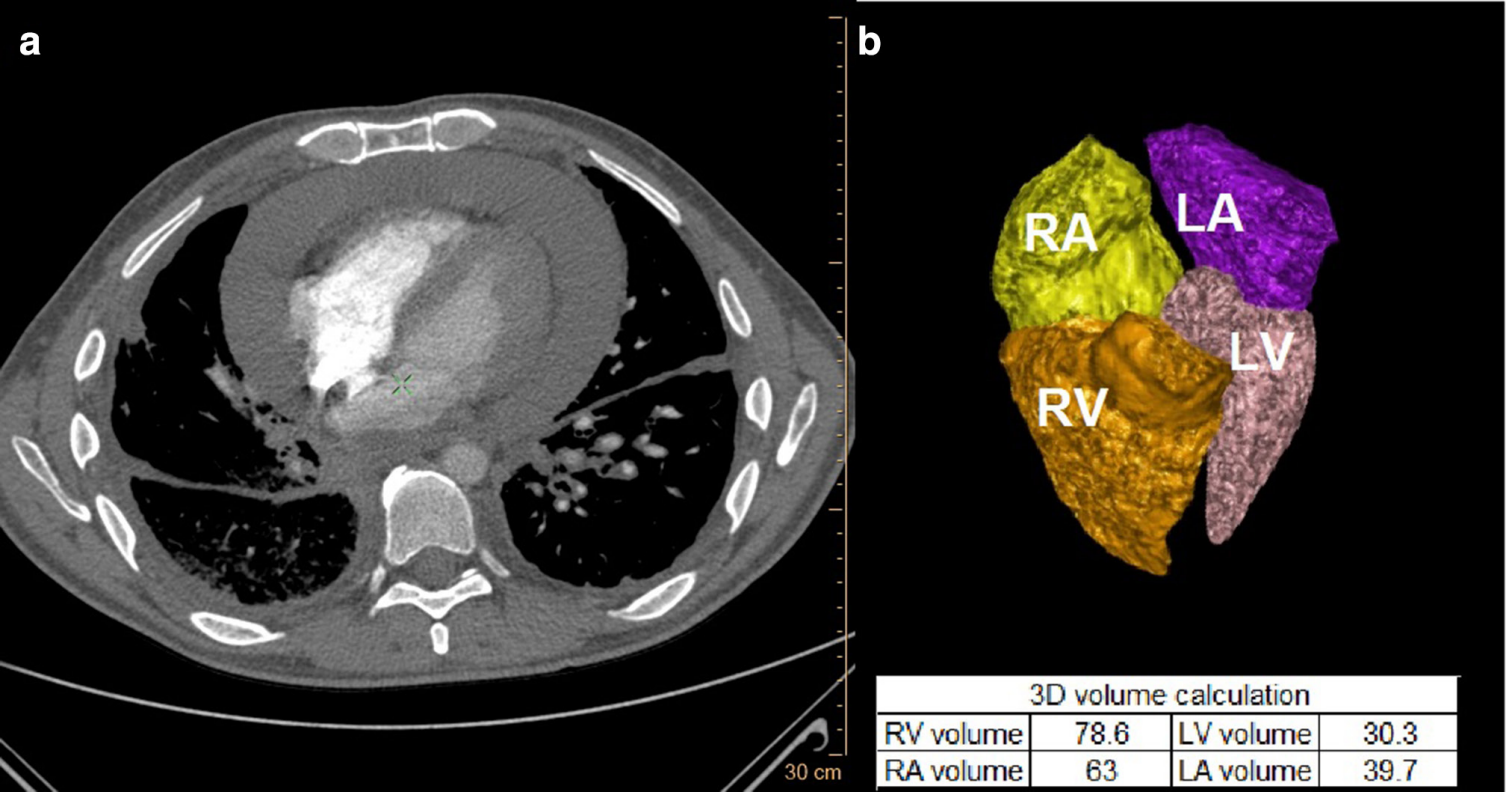
Representative case. Volumetric analysis of the CT pulmonary angiography (CTPA) of a 50-year-old male with a history of lung adenocarcinoma, who presented to the emergency department with shortness of breath. CTPA ruled out pulmonary embolism, but there was a large pericardial effusion. His volumetric analysis was LV volume 30.3 ml, LA volume 39.7 ml, RV volume 78.6 ml, and RA volume 63. Echocardiography showed a moderate degree of pericardial effusion with signs of hemodynamic significance effusion. The patient underwent urgent pericardiocentesis with the removal of 1 l of fluid from the pericardial cavity.

Figures 3 and 4 are representative cases of volumetric analysis of the CT pulmonary angiography (CTPA) of a patient without and with HsPE.

## Discussion

The major finding of this study is the ability of CT-derived cardiac chamber volumes to predict the presence of HsPE with a very good discriminative value. All cutoff values showed a high NPV, thus allowing the radiologist to safely rule out causes of a pending tamponade. PE is caused by the accumulation of fluid in the pericardial sac, with clinical manifestations ranging from asymptomatic, dyspnea, and non-specific chest pain to the most significant of them, cardiogenic shock. These patients could present to the emergency room with symptoms similar to APE and undergo CTPA that will rule out APE but diagnose the existence of the PE.

While the effusion size is an important parameter in evaluating PE, it does not evaluate the clinical and hemodynamic significance of the fluid in the pericardial sac. The relationship between the pericardial volume and pressure is non-linear, whereby a relatively small PE that accumulates rapidly may reduce cardiac filling, rapidly leading to hemodynamic shock, while a chronic very large PE may be observed as having minimal hemodynamic effect^17^ The primary pathophysiology is compression of all cardiac chambers, especially the low-pressure right-sided chambers, as a result of the increased pericardial pressure. This process impairs diastolic filling that in turn decreases stroke volume and cardiac output.^6,7,20^

Echocardiographic assessment of PE is based mostly on the hemodynamic effect of the fluid, such as right heart diastolic chamber collapse when pericardial pressures exceed intracardiac pressure and inspiratory septum “bounce”. However, echocardiography lacks the ability to accurately determine the right chambers’ volumes. CTPA, on the other hand, allows simultaneous full visualization of all four chambers as well as the quantification of their volumes without the need for any geometrical assumptions.^15–18^

Two additional studies used volume analysis of CT scans in patients with pericardial effusion. The first by Ebert et al^21^ showed a method that allows an accurate assessment of the pericardial fluid by CT compared with autopsy report in a specific study population of patients with tamponade caused by rapid accumulation of the fluid (acute trauma or rupture after myocardial infarction or aortic aneurysm) . The second by Liu J et al^22^ utilized a software that quantifies the pericardial effusion size and also gives an estimated HU density (for prediction of hemopericardium). Both studies are limited by the focus on the pericardial effusion size and not on the hemodynamic effect on the cardiac chambers.

Our study findings suggest multiple cutoff values for each chamber, with an RA volume ≤82 ml as being the best predictor of HsPE. The application of a four-chamber volumetric analysis tool is quick and can be executed automatically immediately following the CTPA scan, without any delay or need to transfer the patient to the echocardiography lab. Our results support the contention that an echocardiographic-based finding of RA collapse due to increased pericardial pressure is both a sensitive as well as an early sign of HsPE.^23^ The RA is the chamber with the lowest pressure, especially during late diastole, making it relatively more susceptible to collapse from increased intrapericardial pressure. Thus, a small RA volume can be taken as a sign of significant pericardial pressure.

This study had some limitations. First, the relatively small number of events limited our ability to perform a multivariate logistic regression analysis. Second, the nature of the retrospective analysis in a single tertiary hospital should be validated on a separate dataset. Third, we used non-gated CTPA for the assessment of cardiac volumes, a methodology that is inferior to gated procedures. Our purpose in doing so was to examine data obtained within routine clinical settings, which is currently based on non-gated CTPA scans. Also, volumetric analysis of non-gated CTPA is an accepted solution in other scenario such as assessing RV to LV volume ratio in patients with pulmonary embolism and is used to provide further prognostic information.^18,24,25^

In conclusion, our results suggest that small cardiac chamber volume, especially an RA of <86 ml which is based on automatic analysis of the CTPA scan, may predict the presence of HsPE in a patient with PE. Using automated software analysis, these data can be available soon after the completion of the exam and may allow more expeditious management of these patients.
